# Current management of treatment-induced bone loss in women with breast cancer treated in the United Kingdom

**DOI:** 10.1038/sj.bjc.6602892

**Published:** 2005-11-29

**Authors:** J E Lester, D Dodwell, J M Horsman, S Mori, R E Coleman

**Affiliations:** 1 Academic Unit of Clinical Oncology, Cancer Research Centre, Weston Park Hospital, Sheffield S10 2SJ, UK; 2 Department of Clinical Oncology, Cookridge Hospital, Leeds LS16 6QB, UK

**Keywords:** breast cancer, bone loss, endocrine therapy, adjuvant therapy

## Abstract

New therapeutic options in breast cancer have improved survival but consequently increase the relevance of late complications. Ovarian suppression/ablation and aromatase inhibitors (AI) in the adjuvant setting have improved outcome, but have clinically important adverse effects on bone health. However, investigation and management of cancer treatment-induced bone loss (CTIBL) is poorly defined with no national guidance. In 2004, a questionnaire was sent to over 500 breast surgeons and oncologists who treat breast cancer within the United Kingdom. The questionnaire evaluated access to bone densitometry and specialist expertise as well as attitudes to investigation of CTIBL and anticipated changes in the use of AI for postmenopausal early breast cancer. A total of 354 completed questionnaires were received, 47 from clinicians not currently treating breast cancer. Of the 307 evaluable questionnaires, 164 (53%) were from breast surgeons, 112 (36%) from clinical oncologists and 31 (10%) from medical oncologists. Although most respondents recognised that CTIBL was the responsibility of the treating breast team, investigations for CTIBL are limited even though most had adequate access to bone densitometry; 98 (32%) had not requested a DXA scan in the last 6 months and 224 (73%) had requested fewer than five scans. In all, 235 (76%) were not routinely investigating patients on AI for bone loss. A total of 277 (90%) felt that their practice would benefit from national guidelines to manage these patients, and the majority (59%) had little or no confidence in interpreting DXA results and advising on treatment. This questionnaire has highlighted clear deficiencies in management of CTIBL in early breast cancer. The development of national guidelines for the management of these patients and educational initiatives for breast teams are urgently required.

Many therapies for breast cancer are associated with cancer treatment-induced bone loss (CTIBL). Of principle concern are ovarian suppression in premenopausal women and aromatase inhibitors (AI) in postmenopausal women, which both cause marked reductions in circulating oestradiol that have significant effects on bone physiology.

The most profound changes in bone mineral density (BMD) occur following induction of the menopause, whether induced by oophorectomy, irradiation, LHRH analogues or chemotherapy. BMD can deteriorate by as much as 4.8% at the lumbar spine in just 6 months (Leather *et al*, 1993) while, even in the presence of tamoxifen, ovarian suppression with goserelin results in an average 8% reduction in BMD over 2 years (Gnant *et al*, 2004).

Several recent trials have suggested the benefit of AI's over tamoxifen for the adjuvant therapy of postmenopausal breast cancer patients (The ATAC Trialists’ Group, 2002, 2005; Goss *et al*, 2003; Coombes *et al*, 2004). As a consequence, long-term adjuvant use of an AI or a tamoxifen-AI sequence is anticipated to replace 5 years of tamoxifen as the standard of care for these patients (Winer *et al*, 2005).

Tamoxifen can preserve bone density in postmenopausal women due to its mild oestrogen agonist action on bone (Love *et al*, 1992) although this does not occur in premenopausal women (Powles *et al*, 1996). All of the AIs have been associated with an increased risk of fractures and bone loss of approximately 2% per year (Lester *et al*, 2005). Over a 5-year period of treatment with an AI, an average of 7–8% loss of bone is estimated, equivalent to an average reduction in *T* score of −1.0 or a doubling of fracture risk (Marshall *et al*, 1996).

The trial with the most mature fracture data is the ATAC (Arimidex, Tamoxifen Alone or in Combination) trial which reported a significantly higher risk of fracture compared to tamoxifen at all time points on study (overall 11 *vs* 7.7%) (Howell, 2003) with the most marked differences in fracture rates occurring between 18 and 24 months (Howell, 2003). Following this the fracture rate in anastrozole-treated women plateaued and subsequently fell on completion of treatment.

In most studies, the effects of AI on bone are confounded by either comparison with tamoxifen or use after pretreatment with tamoxifen. Only one study has monitored the effect of exemestane on bone and compared the outcomes to those taking placebo (Lonning *et al*, 2005). This study suggested the effects, at least with this particular AI, were modest and reversible. After 2 years the mean annual rate of BMD loss in the exemestane group was quite similar to placebo in the spine (2.17 *vs* 1.84%, *P*=NS) although significantly greater at the hip (2.72 *vs* 1.48%; *P*=0.024). On withdrawal of exemestane on completion of treatment there was at least partial recovery of BMD over the subsequent year. At present it is uncertain whether the anabolic effect of exemestane will compensate for the class effect of the potent AI on circulating (and tissue) oestradiol levels, and be less detrimental to bone than anastrozole and letrozole. Trials directly comparing the efficacy of the different AI will hopefully clarify this issue.

Guidelines for the management of breast cancer patients at risk of osteoporosis have been published by the American Society of Clinical Oncology (ASCO) (Hillner *et al*, 2003). However, there are no national guidelines in the UK and little or no understanding of the current management of CTIBL in this country.

In the UK, the decision on which endocrine therapy to offer the patient is guided by protocols agreed by the multidisciplinary team (MDT) but the final decision left to the specialist who first sees the patient following surgery. This is often the surgical team, particularly in patients who do not need to see a clinical oncologist to discuss radiotherapy. Patients due to receive chemotherapy will be offered an endocrine therapy by their oncologist once their course of their treatment is completed.

Similarly, follow-up for patients with surgically treated breast carcinoma is generally shared between the oncologists and surgeons. Typically those managed by endocrine therapy alone are followed up by surgeons, while those requiring chemotherapy followed up by oncologists or participate in a shared care arrangement between the two specialties. Thus, all clinicians in the MDT involved in need to be aware of the issues surrounding CTIBL.

In order to facilitate development of UK guidelines we have collated through a questionnaire survey the opinions and understanding of breast cancer specialists on this increasingly important issue to facilitate development of a management strategy for breast cancer women receiving adjuvant treatments.

## MATERIALS AND METHODS

From an internet-based database, a total of 539 breast surgeons and oncologists were highlighted as presently treating patients with breast cancer. Anonymous questionnaires were sent with a covering letter and prepaid return envelope to each of these specialists in May 2004. Further questionnaires were sent to nonresponders in July 2004. The full questionnaire is available on request from the authors.

The questionnaire asked specific questions about the number of breast cancer patients treated per year. Each specialist was asked if they had access to DXA scanning and an osteoporosis specialist, the waiting times for a scan and an estimate of the number of scans performed in the past 6 months. The presence or absence of local guidelines for the monitoring of bone density in breast cancer patients was also determined. Confidence in interpreting DXA scan results and acting upon results was requested. The percentage of patients that are currently prescribed an AI in the adjuvant setting and predicted use in 2–3 years time was ascertained. Specialists were also asked their management of various clinical scenarios associated with CTIBL including both AI use and ovarian suppression. This provided information on the awareness of specialists and the degree of interest in investigating patients. In terms of patient management, specialists were asked which treatments they would offer to patients taking an AI or receiving ovarian suppression with varying levels of BMD (normal, osteopaenia, osteoporosis). The time points when specialists would request repeat DXA scans were also asked. Finally, we asked if specialists felt that their practice would benefit from national guidelines for the diagnosis and management of bone loss in early breast cancer patients.

Questions related to confidence in interpreting scan results or interest in investigating patients in a given clinical scenario were answered using a 5-point scale from 1 (not at all confident/not at all interested) to 5 (very confident/very interested). For some questions these scores were then summarised into low (1 and 2), intermediate (3) and high (4 and 5) for the purposes of reporting and analysis (the full questionnaire is available on line/on request).

## RESULTS

Out of 539 specialists, 354 (66%) returned the questionnaire; 307 were currently treating breast cancer. Of these 307 questionnaires, 164 (53%) were from breast surgeons, 112 (36%) from clinical oncologists and 31 (10%) from medical oncologists.

### Access to and waiting times for DXA

Access to bone densitometry was generally good with 245 (80%) of specialists having a hip/spine or a peripheral DXA machine either locally or within their hospital. The remaining 62 (20%) either did not know or did not have access to a bone densitometer. Many specialists were able to obtain a DXA scan within 12 weeks, however, almost 20% of specialists aware of the waiting times were already experiencing delays of more than 6 months.

### Access to osteoporosis specialist

Only 52 (17%) of specialists had local guidelines for the screening and management of breast cancer patients for bone loss. Most specialists had ready access to an osteoporosis/bone health expert, however, 111 (36%) did not.

### Responsibility for monitoring CTIBL

Specialists were asked who should be responsible for the monitoring of patients at risk from bone loss. Choices included; general practitioner, oncologist, breast surgeon and osteoporosis specialist.

Oncologists were considered to be the most appropriate specialty to take responsibility for monitoring and treating bone issues with 174 (57%) believing that they should be responsible for monitoring and treating the bone effects of adjuvant therapies. This was largely independent of the respondents specialty with 91 (55%) of breast surgeons and 60 (54%) of clinical oncologists recommending follow-up by an oncologist. Slightly more medical oncologists (23, 74%) recommended oncological follow-up.

In all, 57 (19%) indicated that the responsibility should be shared between the breast surgeon and oncologist. Only 43 (14%) of specialists considered that management was the responsibility of the general practitioner.

### Number of scans requested

Monitoring of bone health is not occurring systematically. A total of 98 (32%) had not requested a single DXA scan in the previous 6 months and 224 (73%) had requested fewer than five scans. A total of 235 (76%) are not routinely investigating patients for bone loss while taking an AI.

### Interpretation of results

**Figure 1:**
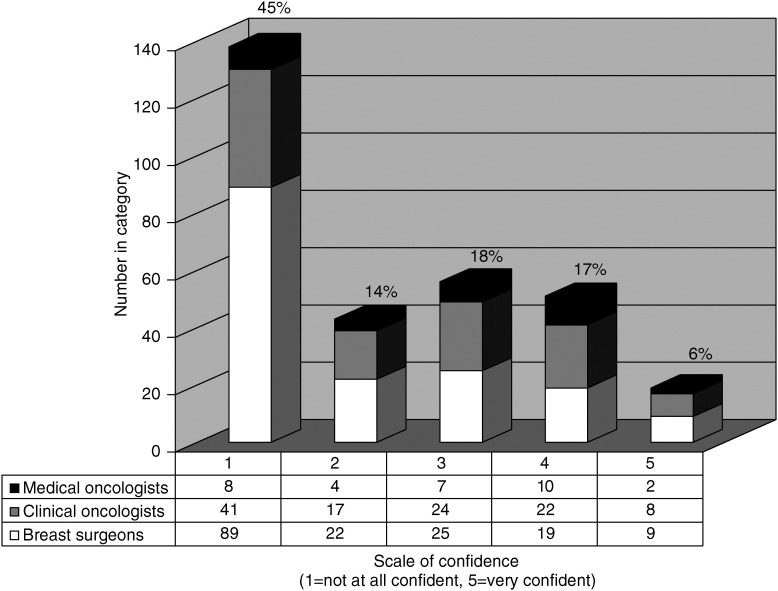
The confidence of breast cancer specialists at the interpretation of DXA based on the question: On a scale of 1–5 how confident are you in interpreting DEXA scan results? (1=not at all confident, 5=very confident).

A question was asked about the confidence of breast cancer specialists in the interpretation of DXA scans. The response was graded from 1 to 5 (1=not at all confident, 5=very confident). Only 70 (23%) expressed confidence with an answer of 4 or 5. A total of 181 (59%) responded with an answer of 1 or 2 and of these 138 (45%) were not at all confident (see Figure 1). Medical oncologists expressed the most confidence with 39% giving a score of 4–5.

### Current and future use of AI's

**Figure 2:**
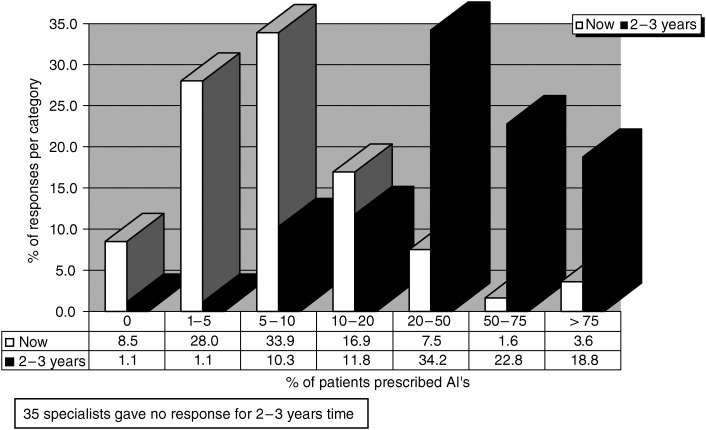
Estimated proportion of patients prescribing AI at the time of the survey and in 2–3 years time based on the question: What proportion of your ER+early breast cancer patients would you prescribe aromatase inhibitors to either instead of tamoxifen or after an initial course of tamoxifen? In all, 35 specialists gave no response for 2–3 years time.

Specialists were asked about the proportion of patients that they prescribed AI's to at the time of the questionnaire and their anticipated use in 2–3 years time. Figure 2 shows that as of 2004 most specialists treat only approximately 5–10% of their patients with AI's. It is clear however that this is anticipated to change considerably over the next few years as treatment guidelines evolve and funding issues are resolved.

### The interest in investigating patients at risk from bone loss

Specialists were asked how interested they would be to investigate various patients on a scale of 1–5 (1=not at all keen, 5=very keen). For the purposes of reporting and analysis, these scores were summarised into a low (1 and 2), intermediate (3) and high (4 and 5) degree of interest. The patients most at risk from future bone loss and the degree of interest in investigating them is summarised in Table 1.

#### Postmenopausal women commencing on an AI

In all, 154 (50%) of specialists responded with low degree of interest in obtaining a baseline BMD value prior to commencing on an AI. Furthermore, only 94 (31%) showed a high degree of interest in investigating these patients.

#### Postmenopausal woman after 2 years of an AI

A greater number of specialists recognised a need for bone densitometry after 2 years of an AI with 141 (46%) recording a response of 4 or 5. However, 89(29%) gave a response of 1 or 2.

#### At 2 years after a premature menopause induced by chemotherapy or ovarian ablation

Specialists were most keen to investigate patients after a treatment-induced premature menopause with 161 (53%) expressing a high degree of interest.

#### Premenopausal woman starting an LHRH agonist for 2–3 years

Few were interested in investigating premenopausal women at the start of treatment with an LHRH agonist with 154 (50%) recording either 1 or 2.

### The treatments and lifestyle advice offered to patients taking AI's

**Figure 3:**
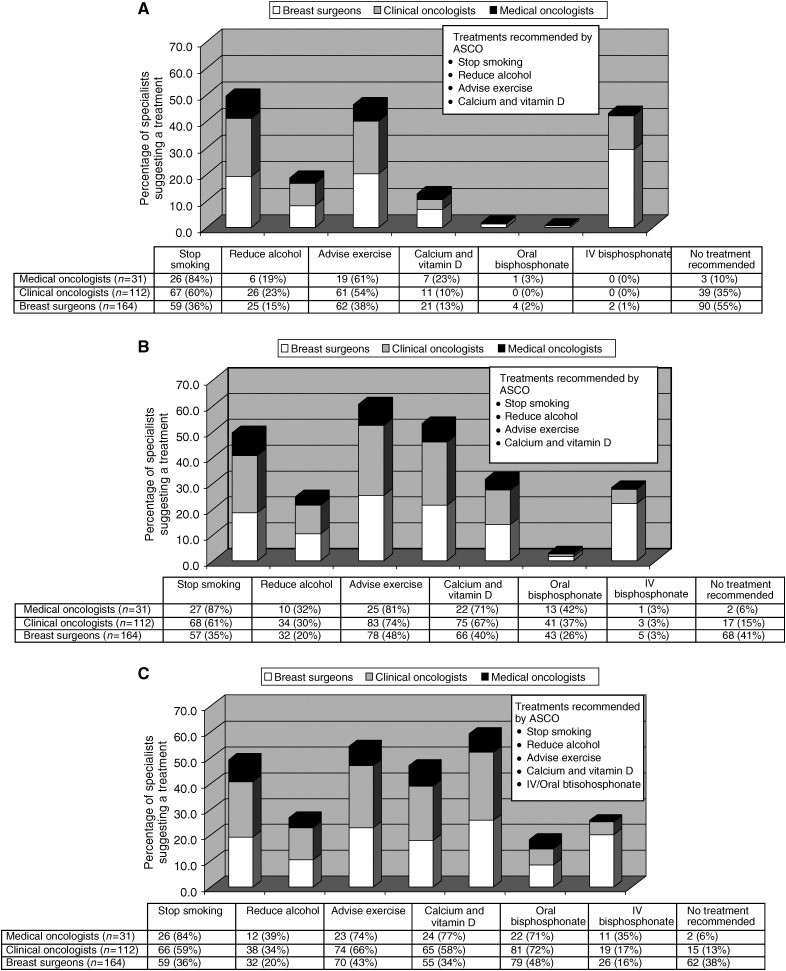
What treatment or lifestyle recommendations would you make in the following clinical situations? (**A**) Patients with normal BMD taking an AI, (**B**) Patients with osteopenia taking an AI and (**C**) Patients with osteoporosis taking an AI.

Figures 3A–C show the percentage of specialists who offered a treatment to patients taking an AI with normal BMD, osteopenia and osteoporosis. Only 39 (12.7%) would prescribe patients calcium and vitamin D supplements to patients with normal BMD. Furthermore, 100 (33%) of specialists did not offer a bisphosphonate to patients with osteoporosis. In all, 79 (26%) of specialists would give no special recommendations to their patients who have been diagnosed with osteopososis while taking an AI. These patients were not offered any lifestyle advice or medications to treat bone loss.

**Figure 4:**
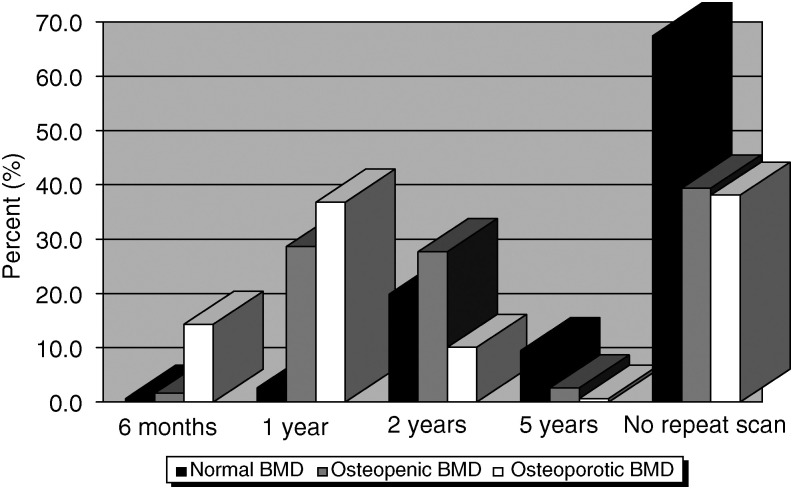
The percentage of specialists who would repeat DXA scans of normal, osteopenic and osteoporotic patients taking AI's when asked: How often would you repeat the DEXA scan in such patients?.

Figure 4 shows when follow-up DXA scans would have been requested in these patients. Only 71 (23%) would repeat a DXA scan after 2 years treatment with an AI if they were found to have normal BMD at baseline.

### Views on the development of a national guideline

Most specialists felt that they would benefit from national guidelines for the management of these patients. In total, 277 (90%) specialists would support the development of a national guideline.

## DISCUSSION

Early reports from clinical trials of AI's *vs* tamoxifen have so far favoured AI's in terms of disease-free survival, risk of contralateral disease and toxicity profile. As a consequence these clinical trials and others still yet to report are likely to account for an increasing use of AI's in the next few years. This increase in breast cancer patients taking AI's, along with increasing use of treatments in young women that result in a premature menopause, is likely to put more women at risk from bone loss and therefore fracture.

This questionnaire has demonstrated that waiting times for bone densitometry is usually greater than 12 weeks, and in some centres patients may wait as long as 6 months. Of specialists, 36% had no access to an osteoporosis expert and only 17% had local guidelines for the screening and management of patients at risk from bone loss. At present breast cancer specialists within the UK request very few DXA scans and only 24% are actively investigating their patients for CTIBL. The ability to interpret DXA scans is also a problem with only 23% expressing a high level of confidence.

Overall the degree of interest in investigating patients at risk from CTIBL is low. The introduction of a national guideline of how to manage CTIBL may increase awareness but also open the door to improved DXA scanning facilities.

Monitoring of CTIBL has implications for the follow-up arrangements for patients with breast cancer. Most specialists consider that the responsibility for monitoring bone loss over the 5–6 years of adjuvant therapy rests with the breast cancer team. However, National Institute of Clinical Excellence (NICE) clinical outcomes guidance recommends discharge from follow-up at 3 years (National Institute of Clinical Excellence, 2002).

In the community, general practitioners are responsible for identifying patients at risk from osteoporosis and will often refer to an osteoporosis specialist for evaluation and advice on management rather than access BMD measurements directly and decide on treatments. Treatment-induced bone loss however, as occurs in breast cancer management, is an uncommon clinical scenario for a general practitioner and, along with issues such as duration of endocrine treatment, likely to be considered the responsibility of the breast team. The development of national guidance in preparation by the National Osteoporosis Society is expected to clarify these lines of responsibility.

It is likely that the sequential use of AI's following a period of tamoxifen will have a less pronounced effect on bone density because of the bone-preserving action of tamoxifen. Estimated bone loss after 5 years of an AI is approximately 7%, however, this may be as little as 2% with tamoxifen-AI sequential therapy. The degree of bone loss expected in a postmenopausal woman over this time period is also approximately 2–3%.

CTIBL is clearly an important issue of international relevance; however, there have been very few published recommendations for the management of these patients (Hillner *et al*, 2003; Coleman, 2004; Lipton, 2004; Theriault, 2004). In 2003, the American Society for Clinical Oncology (ASCO) published a guideline for the monitoring and treatment of bone loss associated with breast cancer treatment (Hillner *et al*, 2003). Calcium and vitamin D supplements were recommended to all patients taking an AI and bisphosphonates for patients with osteoporosis. Educational needs, availability of DXA facilities and clinical protocols are likely to vary from country to country but common principles exist and informal discussion with breast cancer specialists in Europe indicate widespread uncertainty on the appropriate management of CTIBL (Coleman, personal communication).

The patients most at risk of bone loss include postmenopausal women taking AI's, premenopausal women taking LHRH agonists and those with a chemotherapy-induced early menopause. These patients should all be offered calcium and vitamin supplements as well as lifestyle advice such as regular exercise, avoidance of smoking and a healthy diet. Patients over the age of 65 years on a tamoxifen-AI sequence and all patients taking an AI should have a baseline DXA scan. Patients with osteoporosis should be treated with a bisphosphonate known to reduce the incidence of osteoporotic fracture and those with osteopenia have their bone densitometry repeated on a yearly basis and treatment introduced for rapid bone loss or development of osteoporosis.

## Figures and Tables

**Table 1:** The degree of interest in investigating patients at risk from bone loss based on the question: On a scale of 1–5 how keen would you be to investigate the following breast cancer patients for possible osteoporosis? (1=not at all keen, 5=very keen)

	**Degree of interest investigating patient (1=not at all interested, 5=very interested)**		
	**1–2**	**3**	**4–5**
Postmenopausal woman commencing an AI	154 (50%)	59 (19%)	94 (31%)
Postmenopausal woman after 2 years of an AI	89 (29%)	77 (25%)	141 (46%)
Two years after a premature menopause	90 (29%)	56 (18%)	161 (53%)
Premenopausal woman about to start 2–3 years of an LHRH agonist	154 (50%)	61 (20%)	92 (30%)

## Supplementary Material

Appendix1/Supplementary Material
