# Supplementary material for: Current management of treatment-induced bone loss in women with breast cancer treated in the United Kingdom
Source: Br J Cancer. 2005 Nov 29;94(1):30–5. doi: 10.1038/sj.bjc.6602892 (PMC2361086; doi:10.1038/sj.bjc.6602892)
Supplement: Appendix1/Supplementary Material [file 94-6602892x1.doc]

# Appendix 1/Supplimentary Material. Breast Cancer and Treatment Induced Bone Loss Questionnaire

# Breast Cancer and Treatment Induced Bone Loss Questionnaire

# Please circle / tick your response

**Who are you?**

- Breast surgeon
- Clinical Oncologist
- Medical Oncologist
- Other

**Do you treat early breast cancer?**

No – Thanks for your time. Please return questionnaire

If Yes how many do you treat?

- 0-20 per year
- 20-50 per year
- 50 – 100
- >100

**What proportion of your ER+ early breast cancer patients would you prescribe aromatase inhibitors to either instead of tamoxifen or after an initial course of tamoxifen?**

**Currently Expect in 2 –3 years time**

- 0% 0%
- 1-5% 1-5%
- 5-10% 5-10%
- 10-20% 10-20%
- 20 – 50% 20-50%
- 50 –75% 50-75%
- >75% >75%

**Do you have local guidelines for the screening and management of breast cancer patients for osteoporosis / osteopenia?**

- Yes
- No

**Do you have access to a bone densitometer locally or in your hospital?**

- Yes Hip / spine DEXA

Forearm / peripheral scanner

- No
- Don’t know

**Do you have ready access to an osteoporosis / bone health expert?**

- Yes
- No

**What is the average waiting time for a bone density scan?**

- < 1 week
- 1-4 weeks
- 4-12 weeks
- 3-6 months
- >6 months
- Don’t know

**On a scale of 1-5 how confident are you in interpreting DEXA scan results?**

(1=not at all confident, 5 = very confident)

1 2 3 4 5

Who do you think should be responsible for monitoring and treating bone effects of adjuvant therapies?

- GP
- Oncologist
- Breast Surgeon
- Osteoporosis specialist
- Don’t know

## **How many DEXA scans have you requested in the last 6 months?**

- 0
- 1-5
- 5-10
- 10-20
- >20

**Are you routinely investigating patients on aromatase inhibitors for osteoporosis?**

- Yes
- No

**On a scale of 1-5 how keen would you be to investigate the following breast cancer patients for possible osteoporosis?** (1=not at all keen, 5 = very keen)

Post menopausal woman with a recent fracture of the wrist

1 2 3 4 5

Post menopausal woman on an aromatase inhibitor for 2 years out of planned 5

1 2 3 4 5

Post menopausal woman on tamoxifen for 2 years out of planned 5

1 2 3 4 5

Post menopausal woman commencing on an aromatase inhibitor

1 2 3 4 5

Any breast cancer patient over the age of 65

1 2 3 4 5

Any breast cancer patient over the age of 60 with a family history of osteoporosis

1 2 3 4 5

Two years after premature menopause induced by chemotherapy or ovarian ablation

1 2 3 4 5

Pre-menopausal woman starting an LHRH agonist for 2-3 years

1 2 3 4 5

**How would you treat the following patients if they were taking an aromatase inhibitor?** (tick one or more as appropriate)

# T score of the lumbar spine / hip

**+ 0.5** (normal) **- 1.6** (osteopaenic) **- 2.6** (osteoporotic)

Advise stop smoking __ __ __

Vitamin C __ __ __

Calcitonin __ __ __

Oral bisphosphonate __ __ __

Intravenous bisphosphonate __ __ __

Calcium and vitamin D __ __ __

HRT __ __ __

Reduce alcohol intake __ __ __

Advise exercise __ __ __

No special recommendations __ __ __

**How often would you repeat the DEXA scan in such patients?**

# T score of the lumbar spine / hip

**+ 0.5** (normal) **- 1.6** (osteopaenic) **- 2.6** (osteoporotic)

After 6 months __ __ __

After 1 year __ __ __

After 2 years __ __ __

After 5 years __ __ __

No plan to repeat __ __ __

**Do you feel your practice would benefit from national guidelines for the screening for osteoporosis / bone loss in early breast cancer patients?**

- Yes
- No
- Don’t know
